# Horizontal seepage failure model and experimental study of damaged sidewalls of seams between geotube dam tubes

**DOI:** 10.1371/journal.pone.0231624

**Published:** 2020-04-16

**Authors:** Wen-Long Mao, Yi-Ming Shu, Ke Gu, Xian-Lei Zhang, Zhen Zhang

**Affiliations:** 1 College of Water Conservancy and Hydropower Engineering, Hohai University, Nanjing, P.R.China; 2 College of Civil and Transportation Engineering, Hohai University, Nanjing, P.R.China; 3 School of Water Conservancy, North China University of Water Resources and Electric Power, Zhengzhou, P.R. China; China University of Mining and Technology, CHINA

## Abstract

The impact of damaged sidewalls at the joints between tubes on dam structures subjected to horizontal seepage is investigated. First, an experimental scheme is designed to test the mode and critical gradient of seepage failure of the soil in the damaged tubes. The effects of various overburden pressures (0, 5, 10, 20, and 30 kPa), hole radii(0.5, 1.0, 1.5, and 2 cm) and soil specimen properties were studied. The test phenomena and the changes in the pore water pressure were used to determine the seepage failure modes and the critical gradients under different conditions. Combined with the modified Terzaghi soil arching theory, a mathematical model was developed for the critical gradient for soil seepage failure. The model fitting curve was in good agreement with the laboratory test results. The critical gradient is independent of the overburden pressure and weakly dependent on the internal friction angle of the soil. The critical gradient increases with the cohesion. For fixed characteristic soil parameters, the critical gradient decreases at a gradually decreasing rate as the radius of the damaged hole increases.

## 1 Introduction

Geotube dam construction technology offers the advantages of low environmental impact, ease of construction, low cost, and high efficiency [1–4]. For this reason, geotube dams have been widely used in the construction of water conservancy and port projects, such as coastal protection, dike construction, beach reclamation and freshwater reservoirs used for estuarine [5–10].

**Fig 1:**
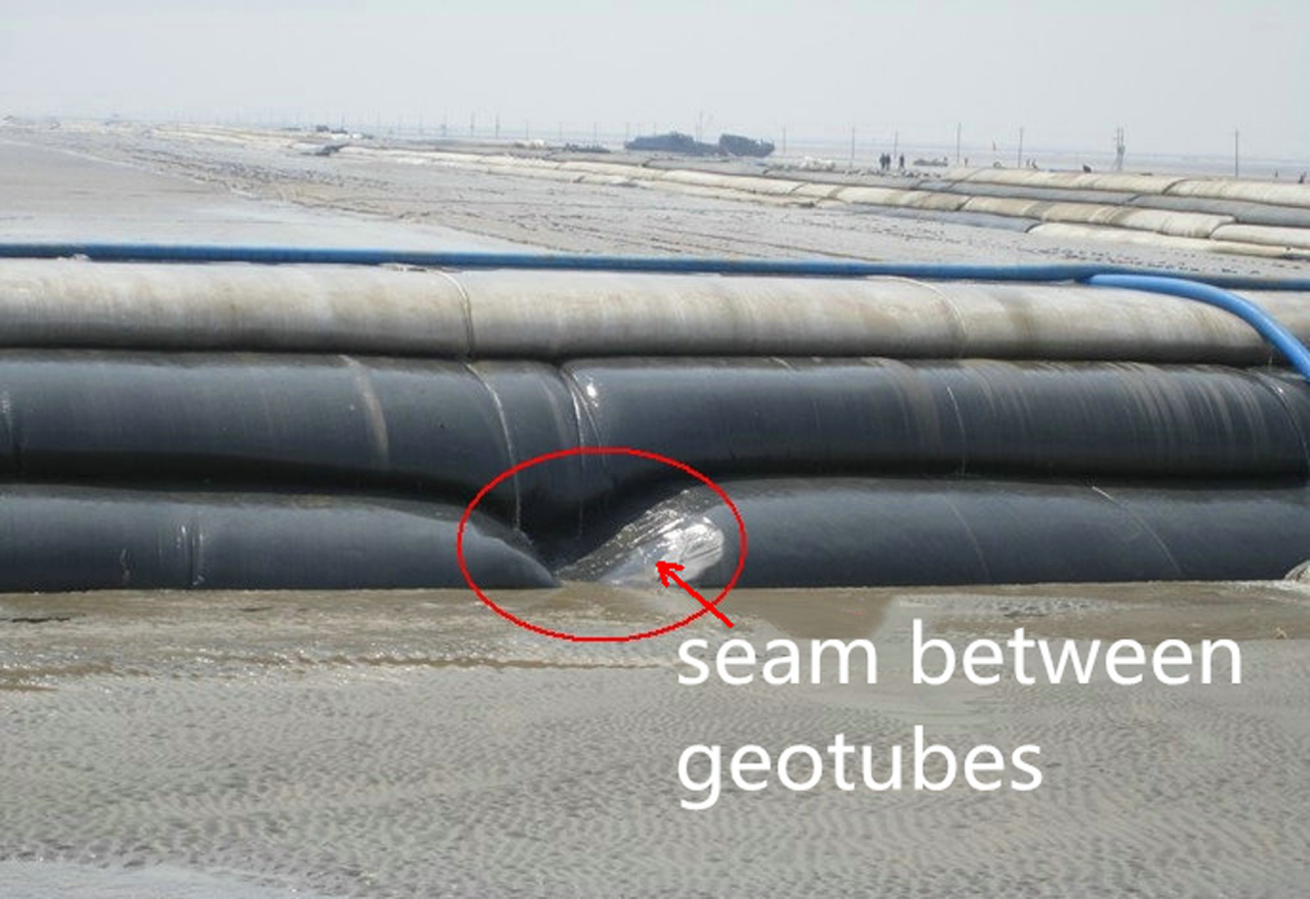
Joint seam between tubes in a geotube dam.

Geotube dams are made of several lapped tubes, with gaps in the lap seams between adjacent tubes, as shown in Fig 1. The geotextile used for geotubes typically meets the basic requirements for drainage and soil retention. Soil in the geotube cannot penetrate the geotextile pores, thereby preventing soil loss [11, 12]. However, the geotextile is occasionally damaged by careless construction or wear and tear[13–17]. Once a reservoir is impounded, the soil in the geotube undergoes seepage erosion under the difference in the hydraulic head between the interior and exterior of the dam[18]. In particular, when the damage is located at the lap seam of adjacent geotubes, the soil in the geotube flows out of the dam through the damaged lap seam, resulting in local dam collapse[19, 20]. For example, in a reservoir used to store freshwater and hold back saltwater that was built in the Yangtze River estuary in the 1990s, the geotube dam underwent large local settlement after more than a decade of operation. Most researchers have concluded from direct observation and analysis that the settlement resulted from the massive loss of soil particles through the seams between the geotubes in the dam[21].

Collapse by seepage failure is a long-term process relative to the service life of most existing projects. Therefore, this problem has not received much attention. Most current studies focus on the structural stability of geotube dams, the filtration and dewatering behavior of the material, and the filling process and late-stage deformation characteristics of the tubes[10, 12, 22–27].

Hence, our research group investigated seepage failure from damage to the walls of the seam between filled tubes. Previous studies have shown that horizontal seepage failure from damaged holes in the sidewall is more severe than vertical upward seepage failure from damaged holes in the bottom wall. Among damaged holes with different shapes and the same area, an O-shaped damage hole presents the least resistance to seepage deformation and failure[28, 29]. Therefore, only the failure of the soil in tubes under horizontal seepage for an O-shaped hole in the seam sidewall was investigated in this study. This study can serve as a useful reference for the design and safety evaluation of practical projects.

First, laboratory tests were performed to determine the horizontal seepage failure modes and critical gradients of soil in damaged geotubes under various operations conditions. Then, a stress analysis of the soil in the damaged hole was performed to develop a mathematical model of the seepage failure gradient. Finally, the test results were fitted using the mathematical model, and the influencing factors were analyzed.

## 2. Horizontal seepage failure test

### 2.1 Test apparatus and materials

#### (1) Test apparatus

**Fig 2:**
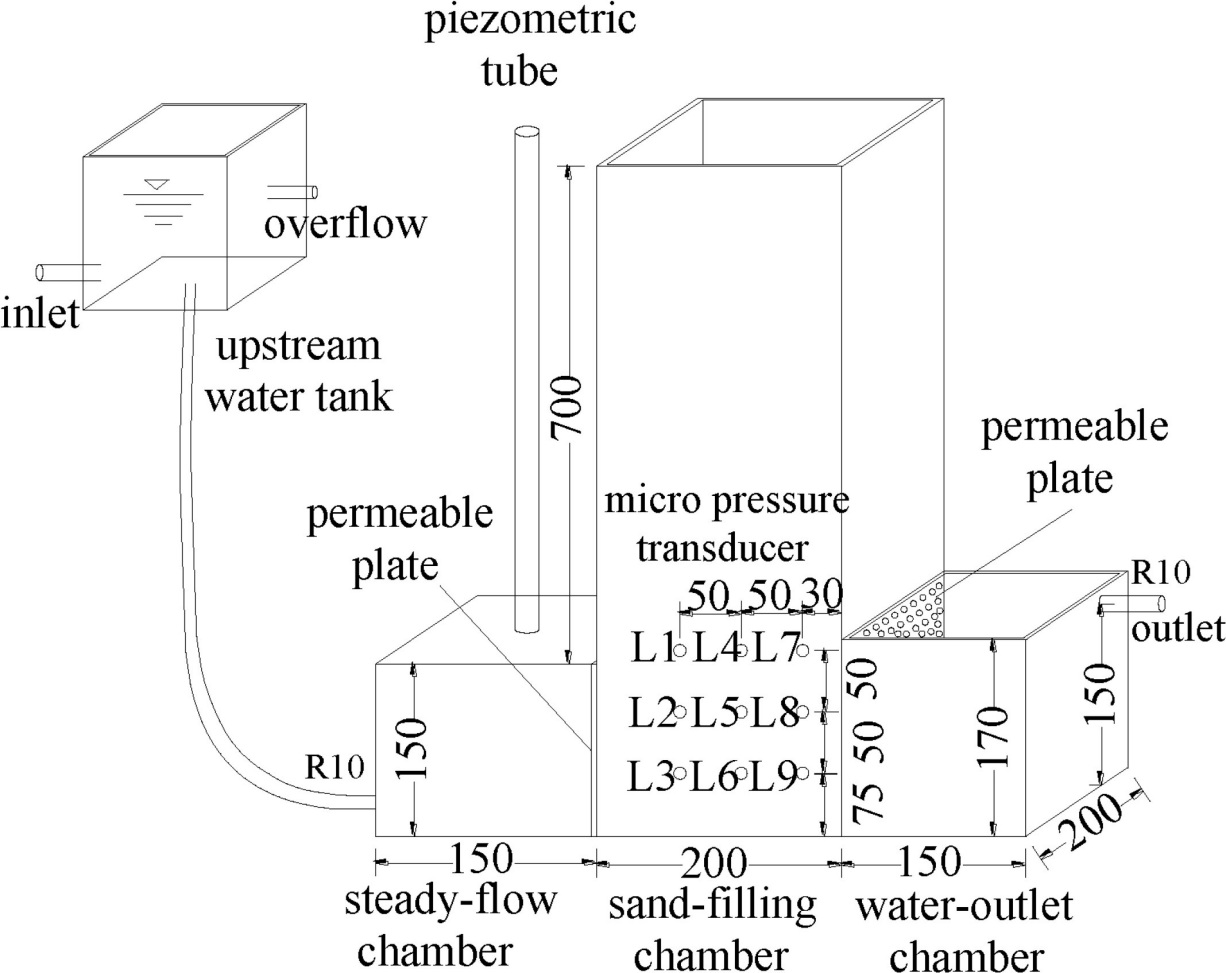
Schematic of apparatus for horizontal seepage failure test (units: mm).

The results of previous soil seepage failure tests were used to design a horizontal seepage failure test apparatus [30–33]. All the chambers were composed of transparent acrylic so that the seepage progress in the soil sample could be observed during the tests. Fig 2 shows the four components of the main body of the apparatus: (left to right) an upstream water tank, a steady-flow chamber, a soil-filling chamber, and a water-outlet chamber.

The upstream water tank is connected to the steady-flow chamber through a hose. An inlet and an overflow port are opened in the left and right sidewalls of the tank, respectively. The inlet is connected to the water supply pump and the overflow port ensures to maintain a constant upstream water level during the test. The steady-flow chamber has net dimensions of 150 mm × 200 mm × 150 mm and provides a steady water flow to the soil specimen. The top center of the steady-flow chamber is connected to a vent tube with a diameter of 20 mm and a height of 700 mm, which also serves as a piezometric tube. The steady-flow chamber and the soil-filling chamber are connected by a porous permeable plate. A geotextile is glued to the plate to prevent soil particles in the soil-filling chamber from entering the steady-flow chamber during the test. The soil-filling chamber has net dimensions of 200 mm × 200 mm × 850 mm. The top of the chamber is left open to enable different overburden pressures to be applied to the soil. A total of nine micro pressure transducers(L1~L9) were placed at predetermined positions to measure the hydraulic pressure upstream(L1~L3), midstream(L4~L6) and downstream(L7~L9) of the seepage field. The measuring range of the transducer is 0~2 kPa, with a maximum error of 0.5%. All the hydraulic pressures were later converted into piezometer heads(unit: cm). The spatial coordinates of the transducer are shown in Fig 2. Hydraulic gradient is calculated by dividing the piezometer head by the corresponding distance. The soil-filling chamber and the water-outlet chamber are also connected by a porous permeable plate glued with a geotextile. A circular 70-mm diameter hole is formed in the center of the plate. This hole was used to make O-shaped holes with different radii, depending on the test conditions, in the geotextileduring the test. The water-outlet chamber has net dimensions of 150 mm × 200 mm × 170 mm, and its top is also left open. A10-mm diameter outlet is opened at a height of 150 mm on the right side to ensure a constant water level in the water-outlet chamber during the seepage process.

#### (2) Geotextile properties

The investigated geotextile is a burst-film woven geotextile that is widely used in hydraulic engineering in China. The main properties of the geotextile are shown in Table 1.

**Table 1:** Properties of woven polypropylene geotextiles.

Property	Symbol	Unit	Value	Standard
mass per unit area	m	g/m^2^	170	-
thickness	T_gt_	mm	0.68	-
coefficient of vertical permeability	k_n_	cm/s	6.7×10^−3^	ASTM D4491
porosity	*n*	-	0.85	-
characteristic opening size	*O*_90_	mm	0.12	ASTM D4751
tensile strength	T_s_	kN/m	75×75	ASTM D4595
ultimate elongation at maximum load	ε_u_	%	17.8×17.8	ASTM D4595
static puncture strength	T_c_	kN	3.4	-

#### (3) Soil properties

**Fig 3:**
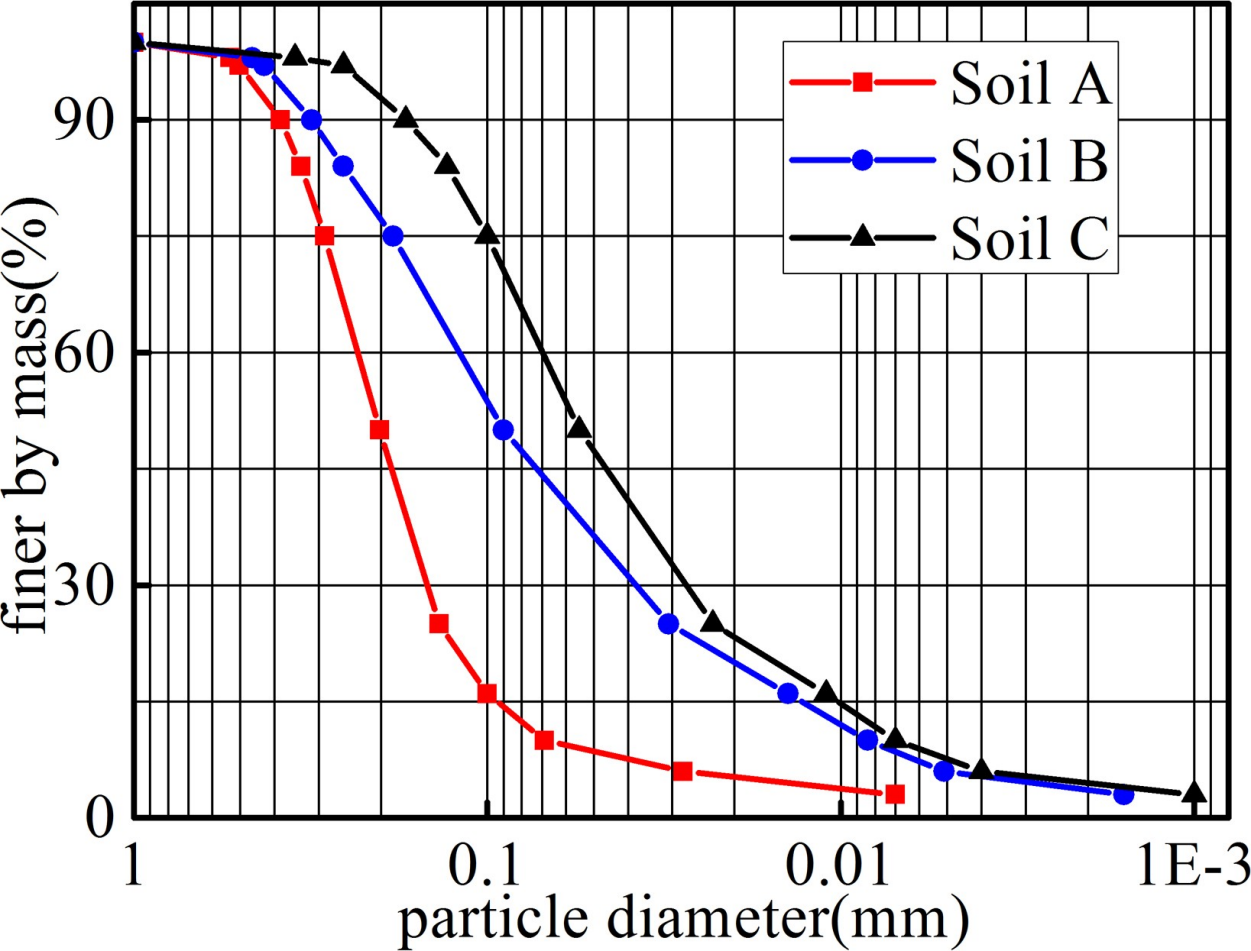
Particle size distribution for test soils.

By mixing silt and sand in different proportions, three artificially mixed soil samples, Soil A, Soil B, and Soil C, were obtained. To ensure a uniform particle size distribution, each specimen was carefully prepared and well mixed. The particle size distribution curves in Fig 3 show that both retention (O_90_ < d_85_) and permeability (O_90_> d_15_) criteria are satisfied. The test results for Soils A, B, and C show internal friction angles of 35°, 33°, and 30°, respectively, and cohesions of 0.5 kPa, 1.0 kPa, and 1.0 kPa, respectively.

### 2.2 Test program and procedure

Based on actual projects in which the cross-sectional diameter of the joint channel is smaller than 5cm, five different hole radii (0.25, 0.5, 1, 1.5, and 2 cm) were investigated for each of the three artificial soil samples. Considering that the overburden pressure varies with the depth of the damaged joint channel between the tubes, four additional overburden pressure levels(5, 10, 20, and 30 kPa) were used for Soil A. A total of thirty-five groups of test results were obtained. A strict procedure (described below) was followed to ensure the repeatability of the experiments.

#### (1) Soil filling and consolidation

Soil was filled in the experiment using the slurry consolidation method. To ensure the homogeneity of the soil sample, the slurry was filled layer-by-layer with a layer height of 5cm. First, the required mass of the 5-cm thick specimen in the soil-filling chamber was calculated according to the minimum porosity. Then, a small amount of water was added to this portion of the specimen, stirred into a homogeneous slurry, and poured into the soil-filling chamber. During slurry consolidation, the soil was continuously compacted with a compaction hammer until the target compactness was reached. This process was repeated until the specimen was filled to the predetermined height. The micro pressure transducers were placed at predetermined positions during soil filling.

#### (2) Specimen saturation

After the soil was fully consolidated, the upstream water tank was raised up, and water was injected into the water-outlet chamber to raise the steady-flow chamber and water-outlet chamber water levels simultaneously. To minimize disturbances to the soil particles from the water flow, the water tank was raised in 2-cm increments and allowed to rest for 1 h after each height increase. After the water level in the water-outlet chamber reached the height of the outlet, water injection was terminated. The soil was soaked for a sufficiently long time under a static head to ensure a high degree of saturation. The entire process lasted for 24 h. For the test group with added overburden pressure, a waterproof shield and overburden load were placed in turn on the top of the soil specimen.

#### (3) Hydraulic head increase

Before each test was conducted, the accumulated soil at the damaged hole was removed to form an exposed vertical soil slope. The hydraulic head was increased in 1-cm increments.The piezometric head and outlet discharge were observed every 1 min at each hydraulic head. The soil specimen was considered stable if there were no changes in the pressure and seepage rate between two consecutive measurements and no notable seepage channels along the sidewall of the apparatus or the soil surface. The hydraulic head was maintained for an additional 20 min and then increased by raising the tank by 1 cm. This procedure was continued until the soil specimen failed completely.

### 2.3 Test results

The same soil under different conditions showed similar failure processes and final failure modes, whereas different soils exhibited significantly different failure modes.

#### (1) Soil A

The seepage erosion process of Soil A can be classified into three stages: stable seepage, initial soil production, and cyclic soil production.

Stable seepage: During this stage, the water in the water-outlet chamber was clear, and no notable changes were detected in the specimen through the transparent acrylic sidewall. With an incremental increase in the upstream hydraulic head, there was a gradual increase in the each piezometric head and the corresponding hydraulic gradient. However, the piezometric head and the corresponding hydraulic gradient under each upstream hydraulic head were stable. These results indicate that the soil specimen remained stable over this hydraulic head range.

**Fig 4:**
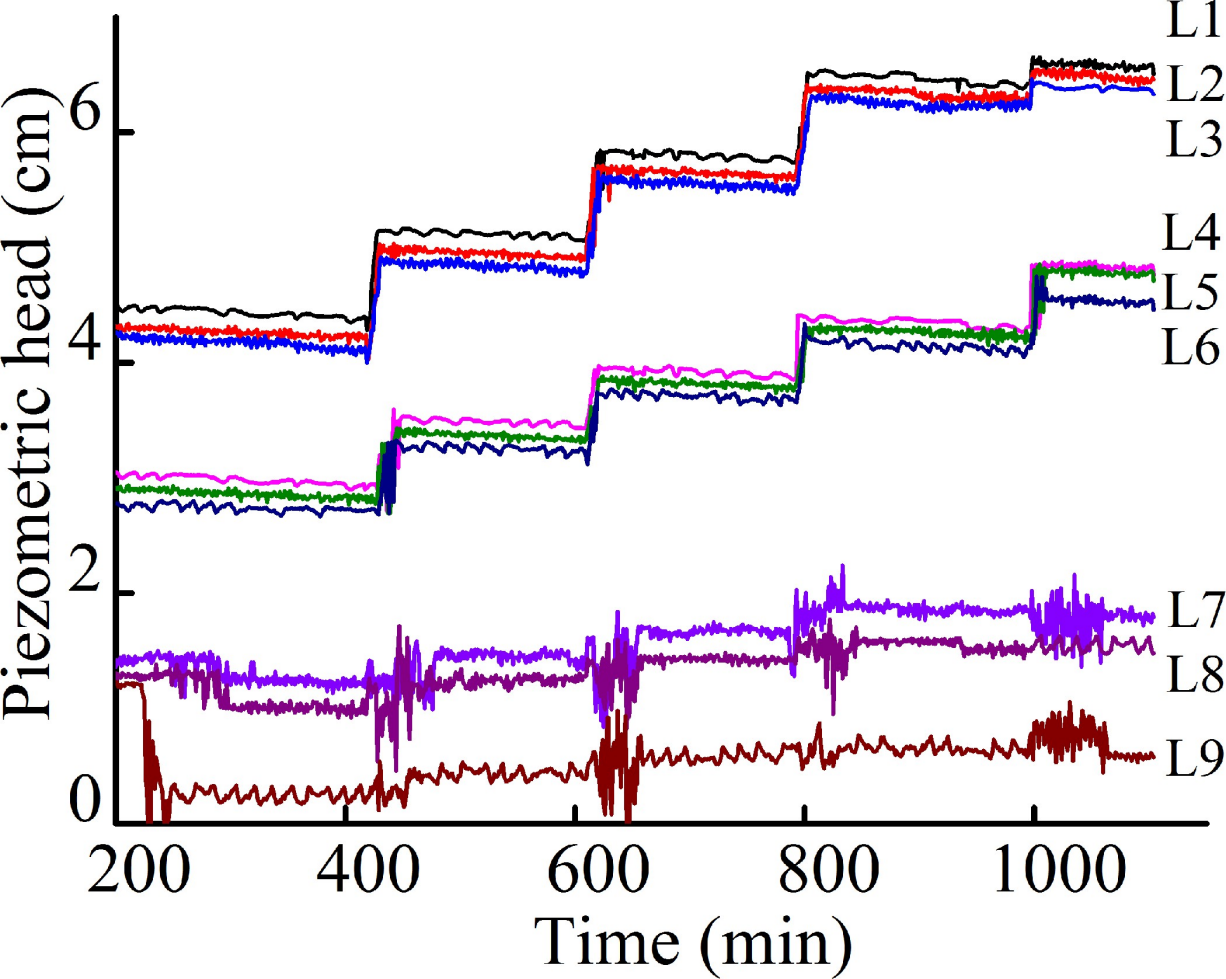
Variation in piezometric head during Soil A test.

Initial soil production: At a certain upstream hydraulic head, the interior soil particles started to wash out through the damaged hole, causing the water-outlet chamber to become slightly turbid. Fig 4 shows the typical variation in the piezometric heads over time after soil production began to occur of Soil A under no overburden pressure and a 1.0-cm hole radius. There was a moderate successive decrease in the pore-water piezometer head at and above the hole(L7~L9), while the piezometric heads at the other positions(L1~L6) did not vary notably. This result indicates soil rearrangement at and above the hole. As the soil washed out increased, most of the soil accumulated at the hole. The hole was gradually submerged, which inhibited and finally terminated soil production. Shortly thereafter, the water in the water-outlet chamber became clear. There was no significant failure was observed, indicating that the soil structure re-stabilized at this hydraulic head.

Cyclic soil production: As the upstream hydraulic head increased, soil production at the damaged hole recommenced, making the water in the water-outlet chamber turbid again. However, soil production terminated as the soil washed out accumulated near the hole. The water in the water-outlet chamber became clear soon thereafter. These results show that the soil structure re-stabilized. Thus, a continuous increase in the hydraulic head produced a characteristic cycle for the water-outlet chamber: soil production- stability and re-soil production.

**Fig 5:**
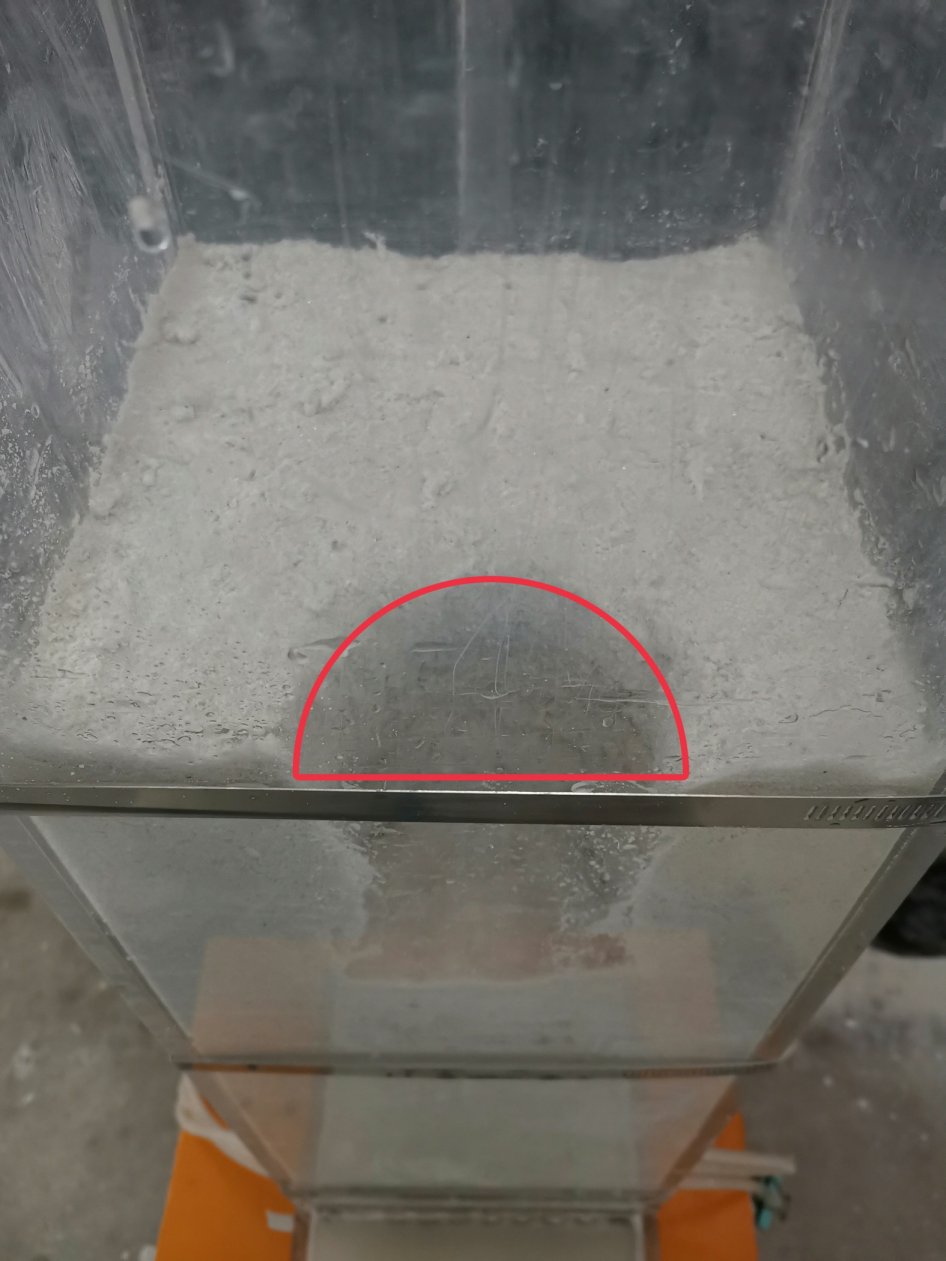
Semicircular collapse pit at top of Soil A.

During this stage, each increase in the hydraulic head caused a corresponding increase in the pore-water piezometer head upstream(L1~L3) and midstream(L4~L6) of the seepage field. The hydraulic heads downstream(L7~L9) did not increase notably, but exhibited significant fluctuations. These changes led to an increase in the hydraulic gradient between the midstream and downstream in the seepage field. However, owing to the gravity of the soil, the failure and readjustment of the soil structure mainly occurred near and above the hole. An arched cavity in the soil above the hole was observed through the transparent acrylic plate. As the upstream hydraulic head increased, the cavity created under each hydraulic head rose until a semicircular collapse pit formed at the top of the soil (as shown in Fig 5). At this stage, the soil structure was considered to have completely failed, and the test was terminated.

#### (2) Soils B and C

Similar seepage erosion processes were observed for Soils B and C. Compared with that of Soil A, there is no difference in stable seepage stage. However, after the initial soil production, the high silt content of the soils resulted in most of the soil washed out being suspended in the water-outlet chamber before flowing out. Only a small portion of soil with large particles accumulated near the damaged hole. Cohesion between fine particles prevented the soil in the soil-filling chamber from collapsing as the soil washed out, and an arched cavity formed instead. The soil accumulation was insufficient to submerge the hole and thereby hindered the soil outflow. Thus, soil continued to flow out of the soil-filling chamber.

**Fig 6:**
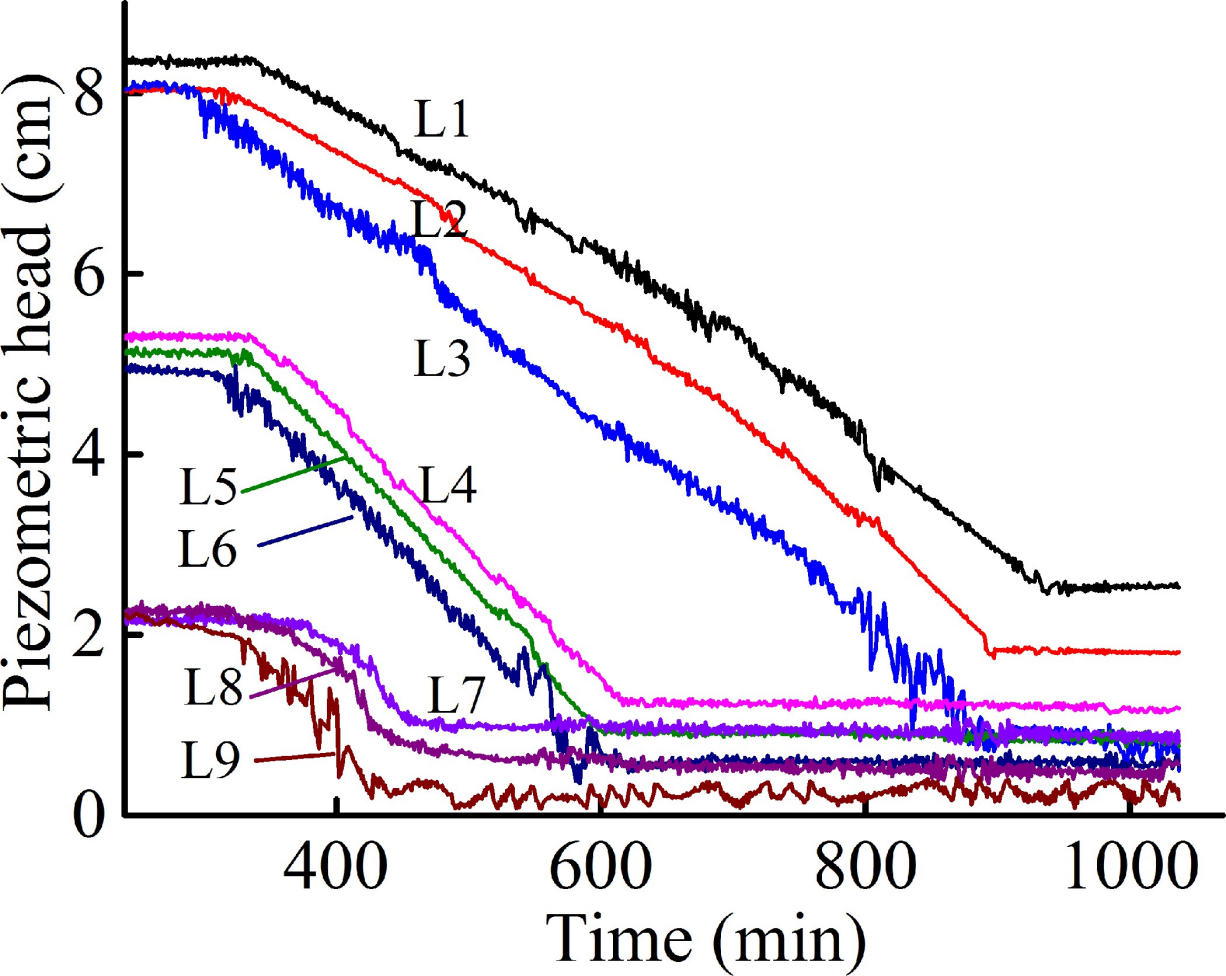
Variations in piezometric head during Soil C testing.

**Fig 7:**
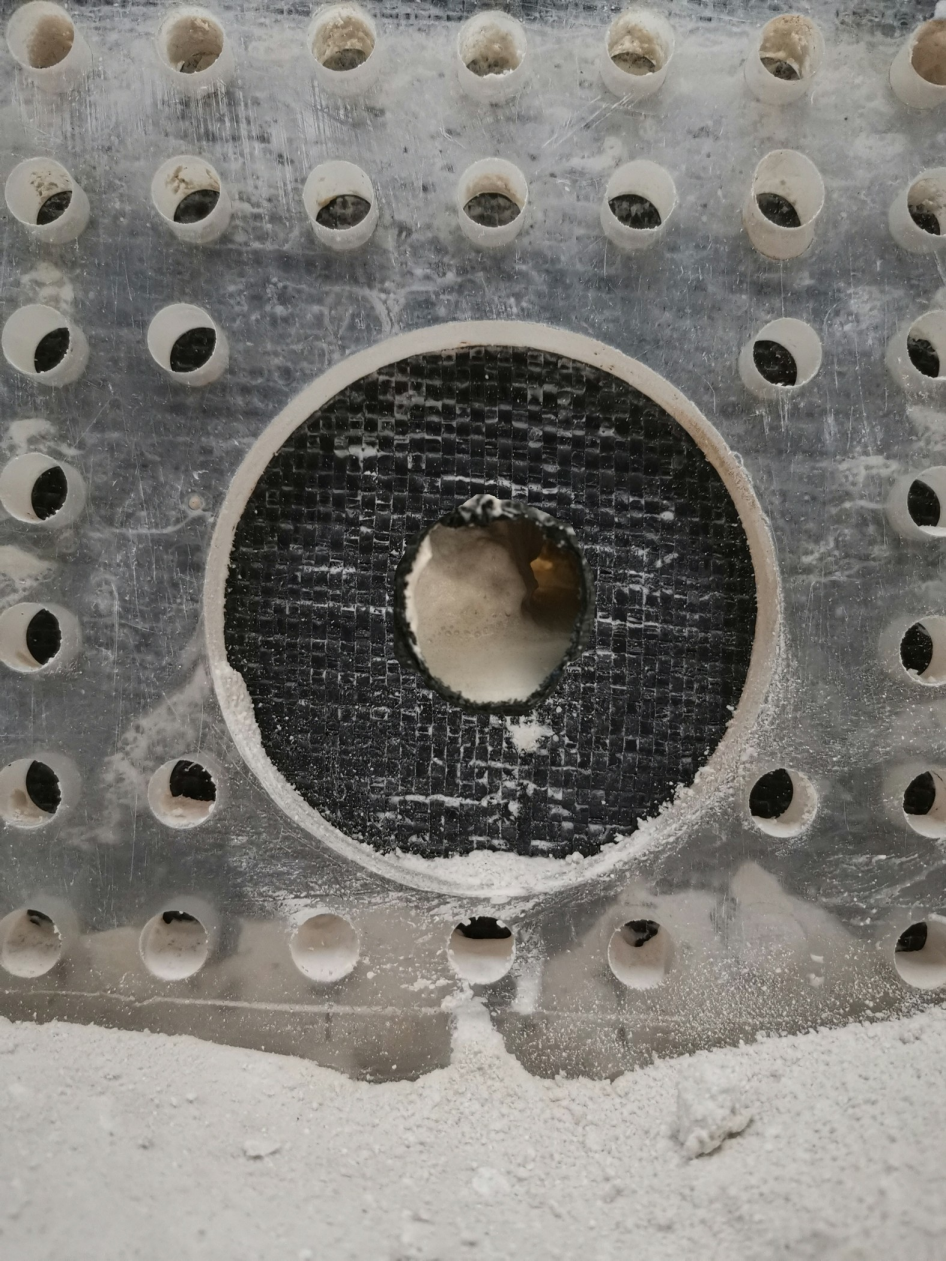
Formation of horizontal soil flow channel after seepage failure of Soil C.

Fig 6. shows the variation in the piezometric heads over time of Soil C after soil production began to occur under no overburden pressure and a 1.0-cm hole radius. The downstream pore-water piezometer head(L7~L9) rapidly decreased to become flush with the water level in the water-outlet chamber. The pore-water piezometer head at other locations (L1~L6) did not vary significantly. This result can be attributed to the excessive loss of particles through the hole, which loosens the soil sample and can even create a cavity near the hole. Thus, the horizontal seepage gradient between the midstream (L4~L6) and the downstream(L7~L9) sections of the seepage field was significantly larger than the vertical gradient between L7 and L9. As seepage erosion progressed, the pore-water piezometer head in the midstream(L4~L6) and upstream(L1~L3) regions successively decreased to a slightly higher level than the water level in the water-outlet chamber. This phenomenon shows that the arched cavity gradually developed upstream along the horizontal direction. At the end of the test, a nearly horizontal soil flow channel formed between the upstream and the damaged hole (as shown in Fig 7).

### 2.4 Critical gradient calculation

**Fig 8:**
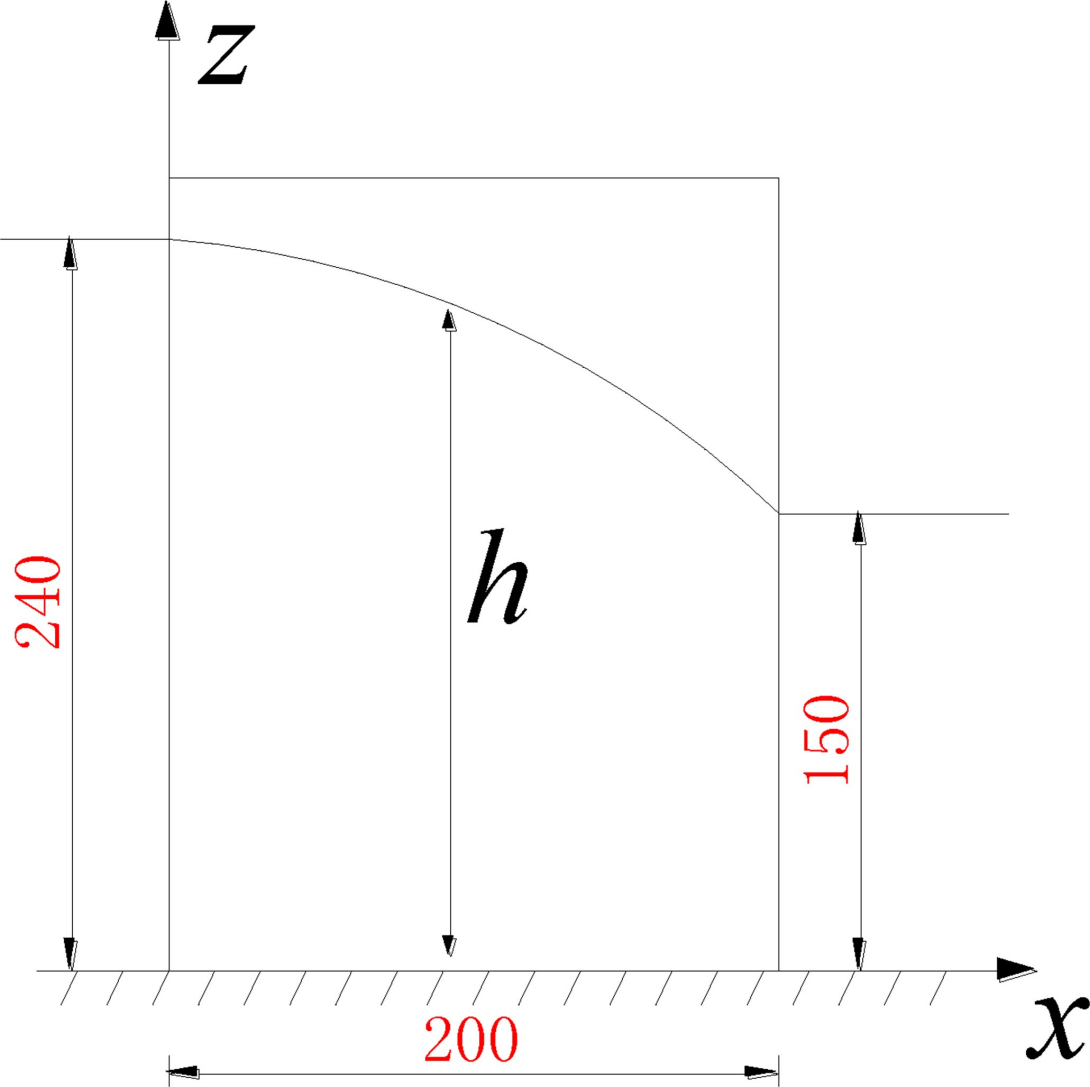
Simplified 2D schematic of seepage field.

Fig 8 is a simplified two-dimensional (2D) schematic of the seepage apparatus in Fig 2. Prior to seepage failure, the steady seepage field for the soil in the apparatus satisfies the seepage continuity equation, namely, the 2D Laplace equation, Eq (1). Under the test boundary conditions, the distribution of the head within the seepage field is given as follows:

$$\frac{\partial^{2}h}{\partial x^{2}}+\frac{\partial^{2}h}{\partial {\text{z}}^{2}}=0$$ (1)

where *h* is the piezometric head.

The seepage field of Soil A before seepage failure under no overburden pressure and a 0.5-cm hole radius is considered as an example. In Fig 8, the upstream and downstream heads in the seepage process are 240 mm and 150 mm, respectively, which satisfy the first boundary condition. The seepage flow at the bottom boundary is zero, which satisfies the second boundary condition. The top curve is the saturation line, which satisfies both the first and second boundary conditions.

The saturation line is determined by polynomial fitting of the piezometric head at different positions and the upstream and downstream water levels. A satisfactory saturation line of *z* = −0.002*x*^2^ – 0.02*x* + 239 is obtained using a quadratic polynomial. Along this line, *h = z*, and the normal derivative of *h* is equal to zero. The complete set of boundary conditions is given in Eq (2):

$$\begin{array}{l} h(0,z)=240\\ h(200,z)=150\\ h_{z}(x,0)=0\\ {h|}_{z=-0.002x^{2}-0.02x+239}=z\\ {\frac{\partial h}{\partial n}|}_{h=-0.002x^{2}-0.02x+239}=0 \end{array}$$ (2)

**Fig 9:**
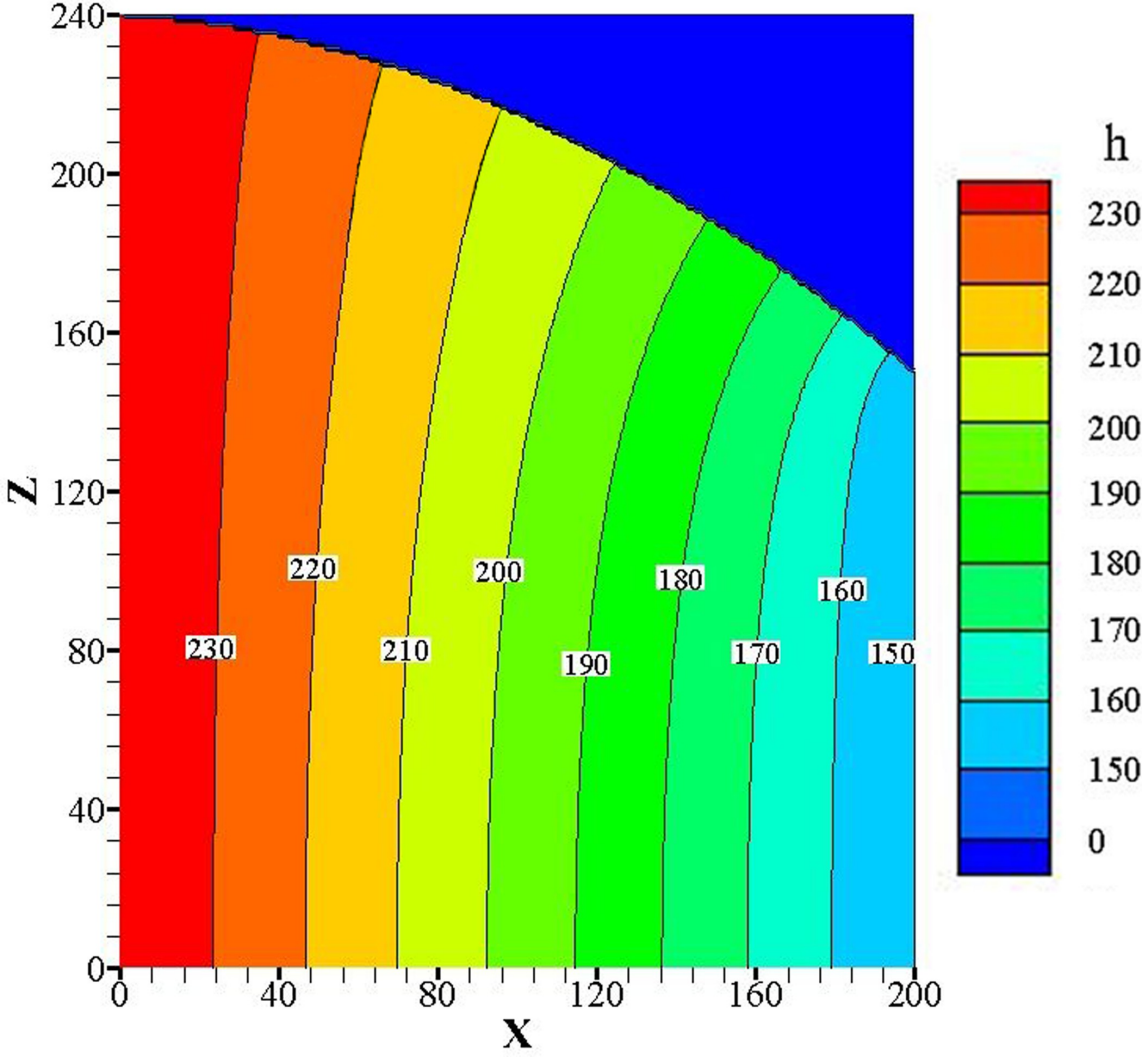
Schematic of equipotential lines in steady seepage.

The complexity of the equations above makes it prohibitively difficult to obtain an exact analytical solution. A Fortran program in Visual Studio was used to obtain a numerical solution. The distribution of the pore-water piezometer head in the seepage field is shown in Fig 9.

In Fig 9, the pore-water piezometer head contours are approximately vertical in the lower portion of the seepage field. That is, for steady seepage flow, the hydraulic gradient direction near the hole is evenly distributed horizontally. As the internal structure of soil has already changed after initial soil production, the hydraulic gradient at this time between the downstream pressure transducer (L7~l9) and the water-outlet chamber can be considered as the critical hydraulic gradient. The critical gradient is calculated by using Eq (3), and the critical gradients under different conditions are summarized in Table 2.

$$J_{cr}=\frac{p_{9}}{l}$$ (3)

Here, *P*_*9*_ is the piezometric head difference, and *l* is the horizontal distance between the L9 pressure transducer and the water-outlet chamber. In this case, *l* = 3 cm (see Fig 2)

**Table 2:** Critical gradient of each group.

Soil type	Soil C	Soil B	Soil A				
Damage hole radius (cm)	Overburden pressure (kPa)						
	0	0	0	5	10	20	30
0.25	2.80	2.74	1.82	1.81	1.84	1.82	1.81
0.5	1.30	1.31	0.81	0.83	0.84	0.82	0.81
1.0	0.70	0.68	0.39	0.37	0.38	0.40	0.36
1.5	0.41	0.43	0.17	0.16	0.17	0.18	0.15
2.0	0.23	0.24	0.10	0.12	0.11	0.10	0.12

## 3. Three-dimensional semicylinder cone-like sliding model

In the stress analysis of the stability of vertical soil excavation, the triangular-wedge model is usually used for theoretical calculations in shield tunneling projects[34]. However, numerous tests in this study showed a semicircular collapse failure mode at the top of the soil (see Fig 5). Therefore, the existing triangular-wedge model was improved and optimized to establish a semicylinder cone-like sliding model.

**Fig 10:**
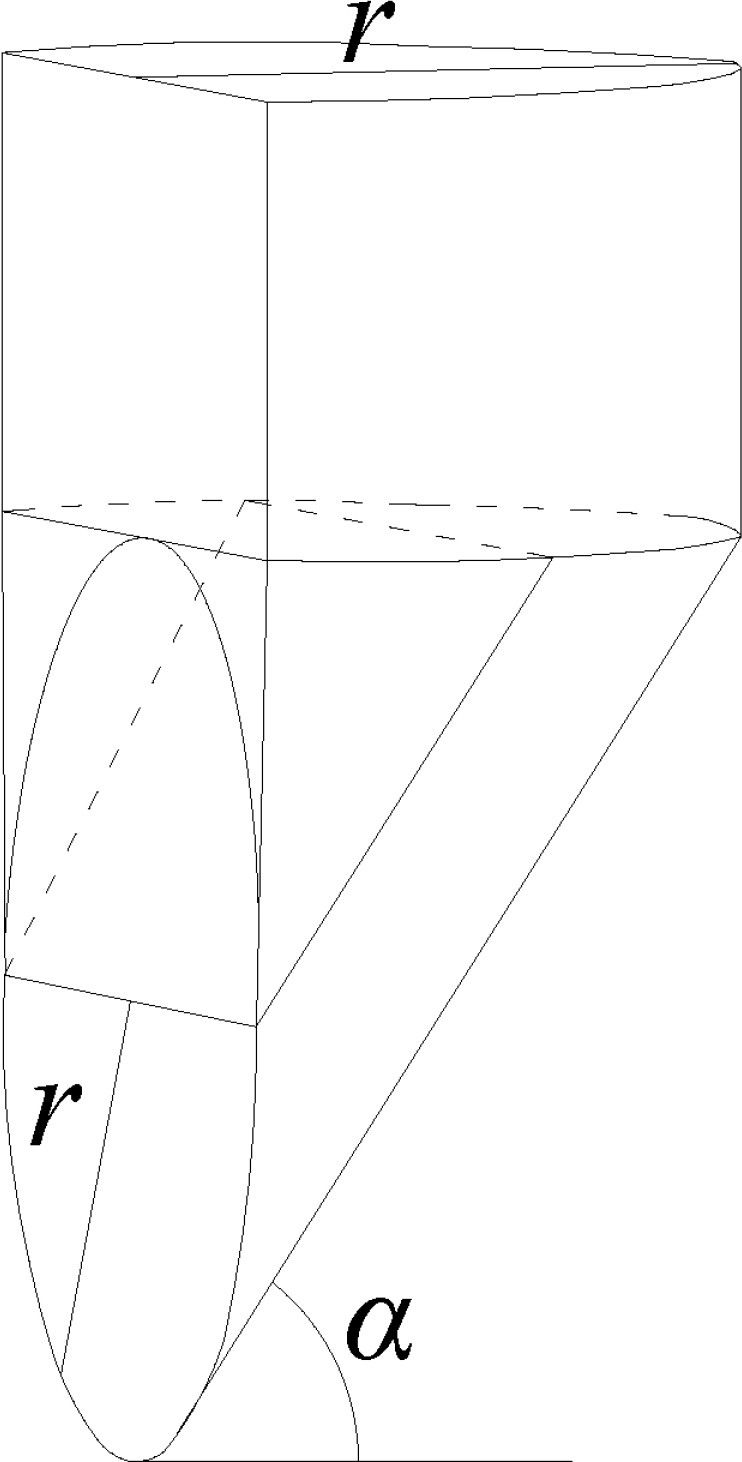
Semicylinder cone-like sliding model.

As shown in Fig 10, the loose soil near the hole is divided into upper and lower regions in the model. The upper region is a semicylinder with a radius equal to that of the damaged hole. The lower region is an irregular cone-like sliding body located in front of the hole. The bottom surface of the sliding body is an arc-shaped sliding surface that forms an angle of $\alpha =\frac{\pi }{4}+\frac{\varphi }{2}$ to the horizontal plane, where *φ* is the internal frictional angle of the soil. The sidewall of the sliding body is the tangent plane of the sliding surface in the vertical direction. The front plane of the sliding body is the vertical surface of the loose soil and is composed of a semicircular damaged hole and a rectangular shape that is externally tangent to the semicircular damaged hole.

### 3.1 Vertical force

To analyze the stress equilibrium of the cone-like sliding body, the earth pressure *σ*_*v*_ from the upper semicylindrical soil is calculated. Some soil washes out through the damaged hole during the filling process, resulting in differential settlement of the upper soil and a subsequent arch in the soil structure. Therefore, the modified Terzaghi soil arching theory can be used for the calculation.

**Fig 11:**
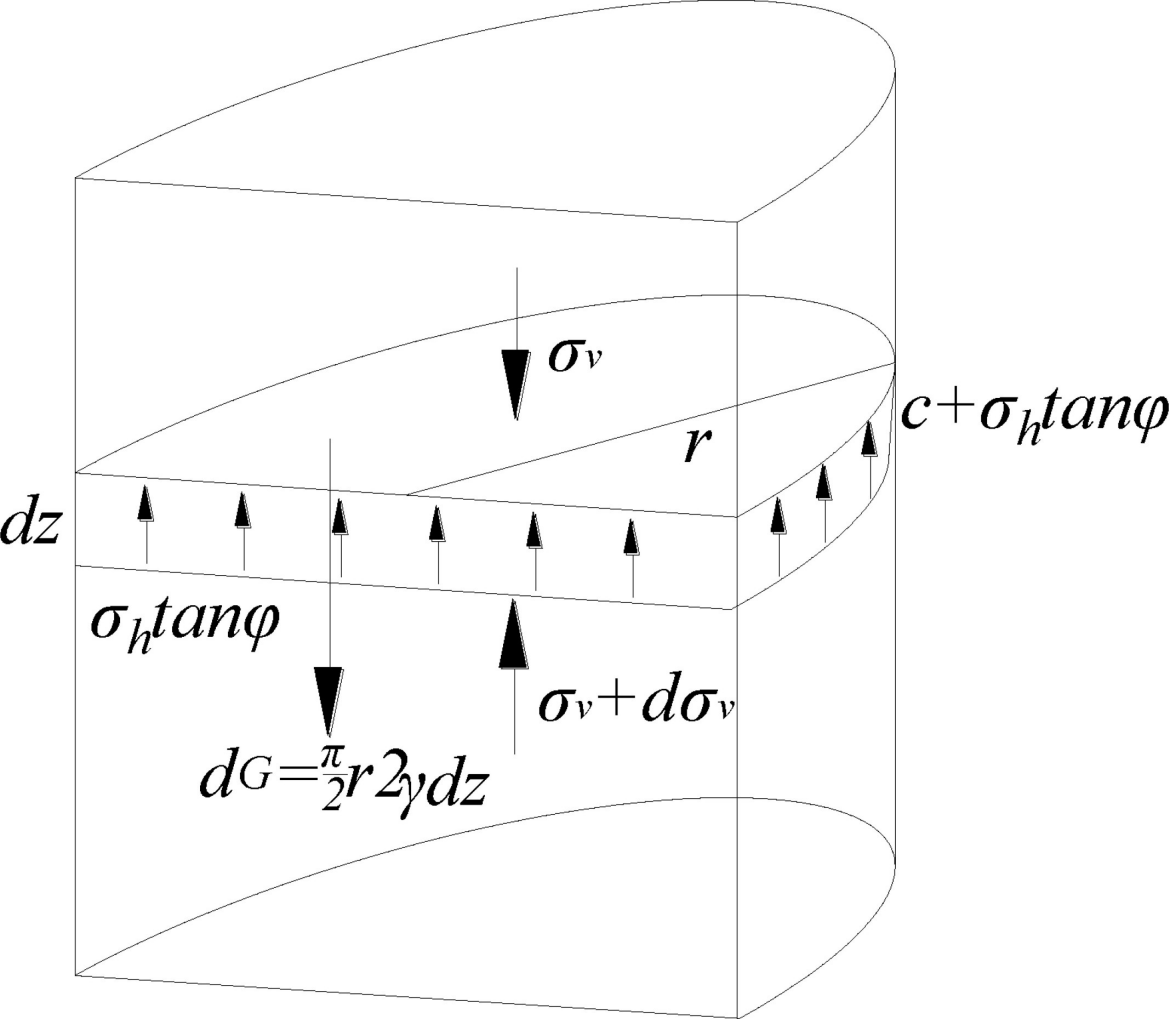
Schematic of modified soil arching theory calculation.

Considering the experimental test results, the stress analysis is performed on a semicircular thin layer soil with a thickness of *dz* at a subsurface depth of *z*, as shown in Fig 11. The stresses acting on the thin layer of soil include the vertical stress *σ*_*v*_ from the upper soil region, support stress *σ*_*v*_ + *dσ*_*v*_ and gravity $\frac{\pi r^{2}}{2}\gamma dz$.

The normal stress *σ*_*h*_ on the vertical sliding surface given by the static earth pressure theory is

$$\sigma_{h}=K\sigma_{v}=(1-\;\sin\;\varphi )\sigma_{v}$$ (4)

where *K* is the coefficient of the lateral pressure of the soil.

The soil shear stress *τ* from Coulomb shear strength theory is

$$\tau =c+\sigma_{h}\;\text{tan}\;\varphi$$ (5)

where *c* is the cohesion of the soil.

Then, the force exerted by the surrounding soil on the thin layer can be obtained as

f=πrτdz (6)

Assuming that the friction coefficient between the soil and the geotextile is equal to the internal friction coefficient of the soil, the friction force exerted by the geotextile on the semicircular thin layer is 2*r σ*_*h*_ tan *φdz*.

For stable soil, there is a zero resultant vertical force on the thin layer. The corresponding equilibrium condition can be written as

$$\frac{\pi r^{2}}{2}\gamma dz-\frac{\pi r^{2}}{2}\left(\sigma_{v}+d\sigma_{v}\right)+\frac{\pi r^{2}}{2}\sigma_{v}-f-2r\sigma_{h}\;\text{tan}\;\varphi dz=0$$ (7)

where *γ* is the soil bulk density.

The equation above is integrated to yield the vertical stress *σ*_*ν*_:

$$\sigma_{v}=\frac{\pi r}{\left(2\pi +4\right)K\;\text{tan}\;\varphi }\left(\gamma -\frac{2c}{r}-c_{2}e^{-\frac{\left(2\pi +4\right)K\;\text{tan}\;\varphi z}{\pi r}}\right)$$ (8)

where *c*_*2*_ is the integration constant.

If a uniform pressure *q* is applied to the soil surface, then *z* = 0, *σ*_*ν*_ = *q*, and

$$\sigma_{v}=\frac{\pi \left(\gamma r-2c\right)}{\left(2\pi +4\right)K\;\text{tan}\;\varphi }\left(1-e^{-\frac{\left(2\pi +4\right)K\;\text{tan}\;\varphi z}{\pi r}}\right)+qe^{-\frac{\left(2\pi +4\right)K\;\text{tan}\;\varphi z}{\pi r}}$$ (9)

When the depth of soil is much larger than the radius of the damaged hole (i.e., *z* ≫ *r*), $e^{-\frac{\left(2\pi +4\right)K\;\text{tan}\;\varphi z}{\pi r}}$ is approximately zero. The uniformly distributed overburden pressure is then negligible, and the vertical stress reaches the following stable value:

$$\sigma_{v}=\frac{\pi \left(\gamma r-2c\right)}{\left(2\pi +4\right)K\;\text{tan}\;\varphi }$$ (10)

This formula is the modified Terzaghi soil arching theoretical prediction, which shows that when *z* ≫ *r*, the vertical stress eventually reaches a constant value. The vertical force *P*_*v*_ can be obtained by multiplying the vertical stress by the area of the top surface of the cone-like sliding body:

$$p_{v}=\sigma_{v}A=\frac{\pi r^{2}\sigma_{v}}{2}$$ (11)

where *A = πr*^*2*^/*2* is the area of the top surface of the sliding body.

### 3.2 Seepage force

In a unit volume of soil, the drag exerted by the seepage flow on soil particles is expressed as follows [35]:

$$f_{x}=-\gamma_{w}\frac{\partial h}{\partial x},f_{y}=-\gamma_{w}\frac{\partial h}{\partial y},f_{z}=-\gamma_{w}\frac{\partial h}{\partial z}$$ (12)

where *γ*_*w*_ is the unit weight of water.

Fig 9 shows that the hydraulic head lines near the hole in the seepage field are vertically distributed. Thus,

$$f_{y}=f_{z}=0$$ (13)

As is shown in Fig 10, the seepage force acting on the sliding body in the x direction is the surface integral of the seepage force per unit volume on the sliding body in front of the hole, that is, [35]

$$F_{x}=\gamma_{w}\;\text{sin}\;\alpha \int_{s_{a}}h^{*}ds$$ (14)

where *s*_*a*_ is an arc-shaped sliding surface of the sliding body, and h* is the piezometric head of the pore-water at the intersection of the hole center and the sliding surface in the *x* direction.

Evaluating the integral in Eq (14) yields

$$F_{x}=\frac{\left(\pi +4\right)r^{2}\gamma_{w}h^{*}}{2}$$ (15)

### 3.3 Model force analysis

**Fig 12:**
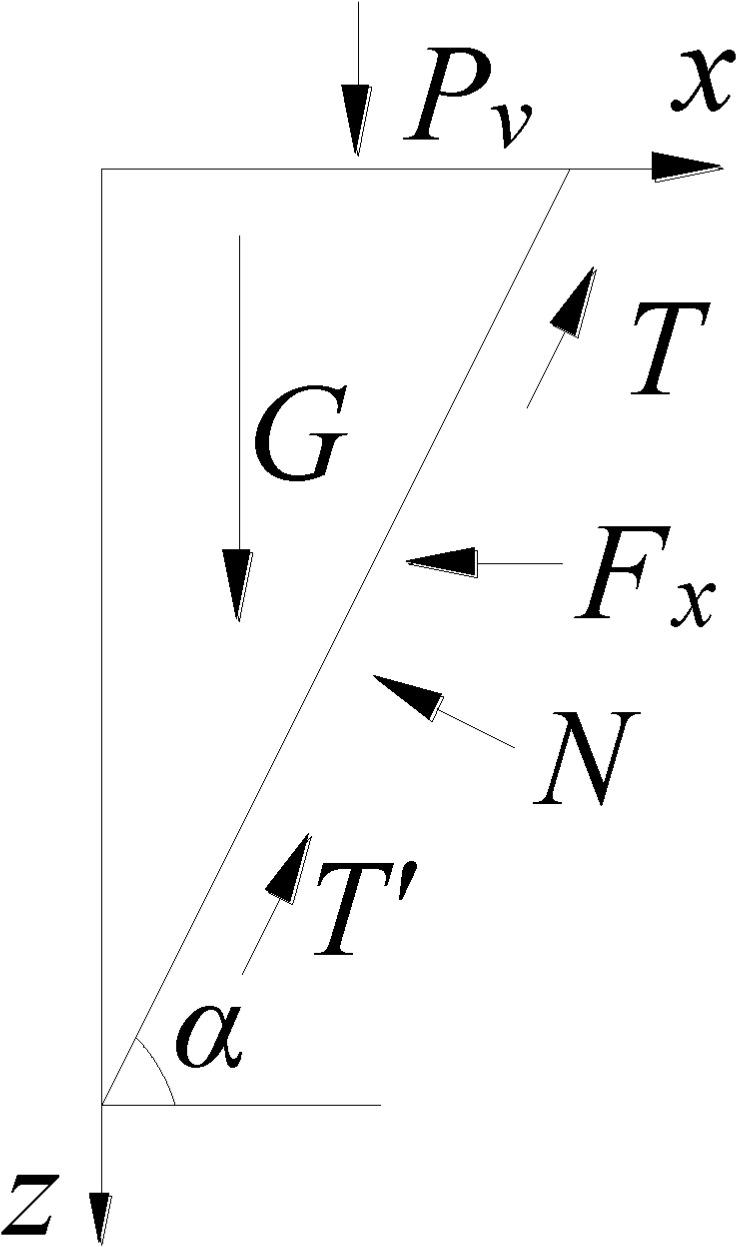
Forces acting upon cone-like sliding body. Symbols: *P*_*v*_ = vertical force; ***F***_***x***_ ***=*** horizontal seepage force; ***G =*** self-gravity; ***T =*** friction at the bottom surface; ***T***^***’***^ = friction at the side surface; ***N* =** bottom surface support force; *α* = angle of inclination of the sliding body.

The cone-like sliding body in front of the damaged hole is subjected to the upper vertical force *P*_*v*_ and the horizontal seepage force *F*_*x*_ as well as self-gravity *G*, friction at the bottom surface *T*, friction at the side surface *T'*, and the bottom surface support force *N*. The force diagram can be simplified to the triangle shown in Fig 12.

(1) The self-weight *G* of the cone-like sliding body is given by:

$$G=\gamma^{\prime }V=\gamma^{\prime }(V_{1}+V_{2}+V_{3})$$ (16)

where *γ'* is the submerged unit weight of soil.

**Fig 13:**
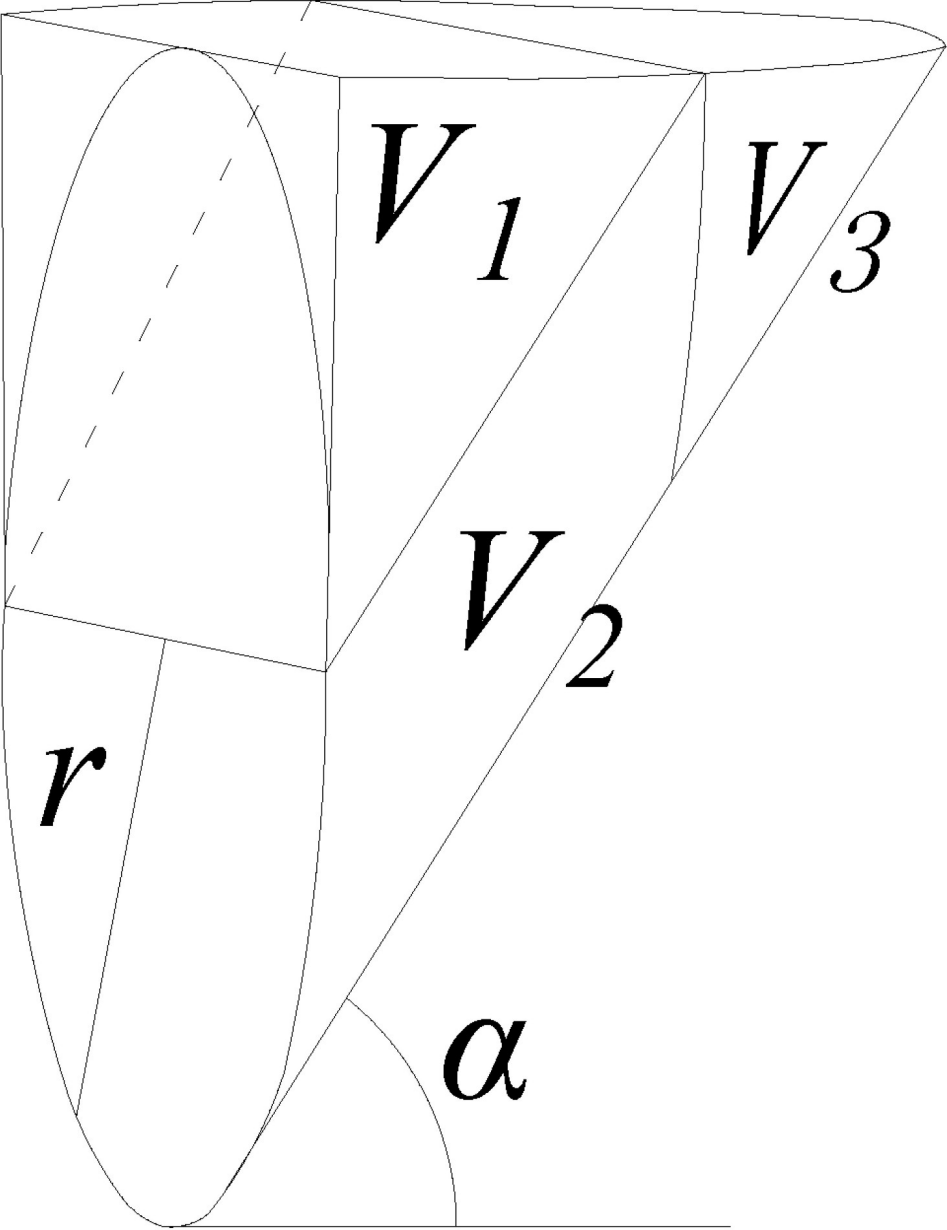
Schematic for volume calculation of cone-like sliding body.

The weight of the cone-like sliding body is calculated by dividing the body into parts. As shown in Fig 13, V1 and V2 are the triangular prism and the oblique circular cylinder of the body, respectively, and their corresponding volumes are calculated as follows:

$$V_{1}=\frac{1}{2}\cdot r\cdot \frac{r}{\;\text{tan}\;\alpha }\cdot 2r;\;V_{2}=\frac{1}{2}\cdot \pi \cdot r^{2}\cdot \frac{r}{\;\text{tan}\;\alpha }$$ (17)

**Fig 14:**
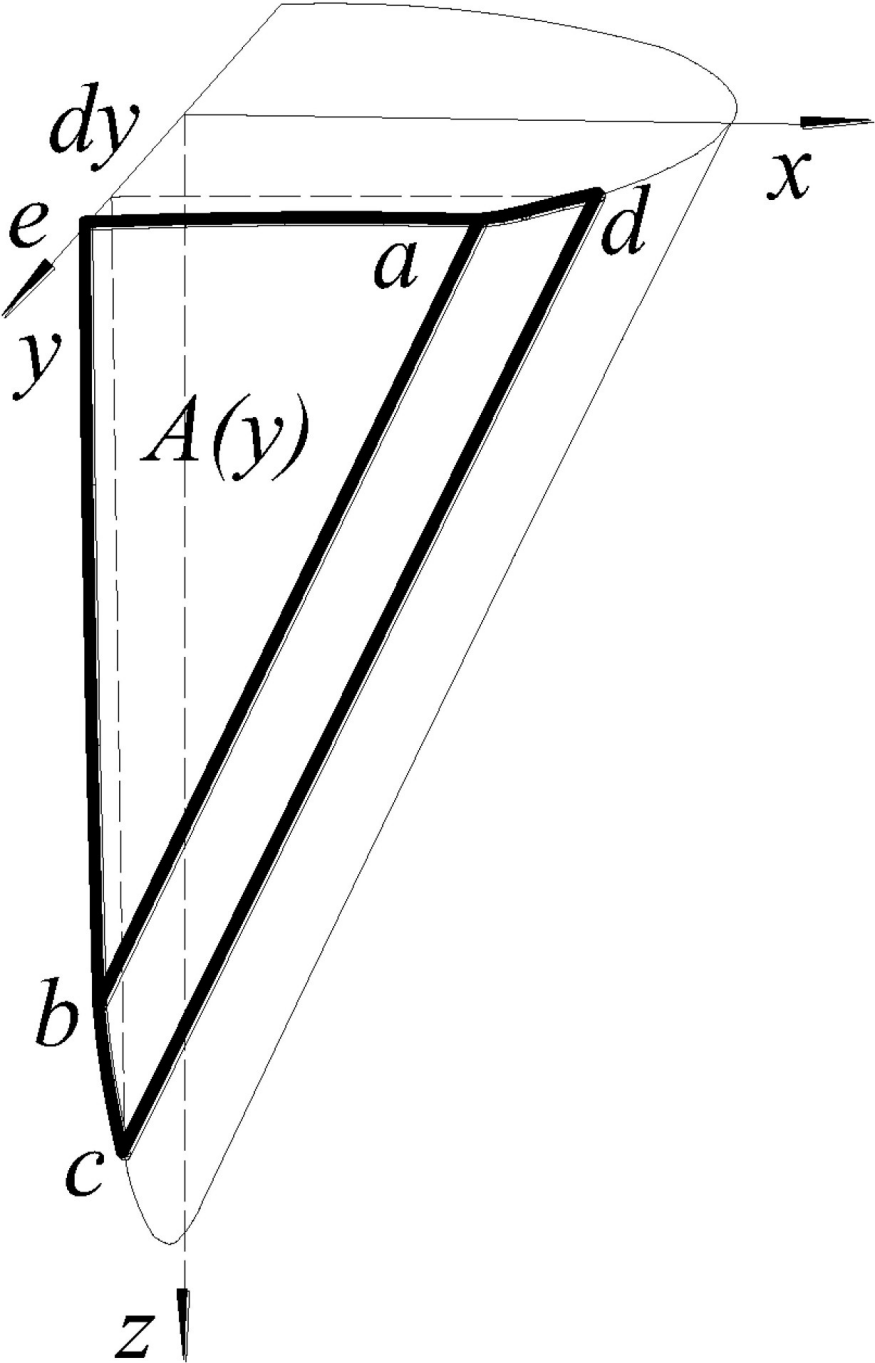
Schematic for wedge calculation.

V3 is the wedge formed by slicing the oblique circular cylinder by a vertical plane, and its volume is calculated by integration of the infinitesimal element *abe* shown in Fig 14:

$$V_{3}=\frac{1}{2}\int_{-r}^{r}\sqrt{r^{2}-y^{2}}\cdot \frac{\sqrt{r^{2}-y^{2}}}{\;\text{tan}\;\alpha }dy$$ (18)

Substituting Eqs (17) and (18) into Eq (16) yields the total weight of the cone-like sliding body as

$$G=\gamma^{\prime }v=\frac{\pi r^{3}\gamma^{\prime }}{2\;\text{tan}\;\alpha }+\frac{r^{3}\gamma^{\prime }}{\;\text{tan}\;\alpha }+\frac{2r^{3}\gamma^{\prime }}{3\;\text{tan}\;\alpha }=\frac{\left(3\pi +10\right)r^{3}\gamma^{\prime }}{6\;\text{tan}\;\alpha }$$ (19)

(2) To calculate the friction *T*, the area of the friction interface at the bottom of the sliding body is also calculated by dividing the body into parts. *S*_*2*_ is the area of the contact surface of the lower portion V_2_ of the sliding body, which is given by the surface area of an oblique circular cylinder:

$$S_{2}=\pi r\cdot \frac{r}{\;\text{sin}\;\alpha }$$ (20)

To calculate the area S_3_ of the contact surface of the upper portion V_3_ of the sliding body, the area of the infinitesimal surface *abcd* (see Fig 14) is calculated and integrated along the *y* direction.

$$S_{3}=\frac{1}{\;\text{sin}\;\alpha }\int_{-r}^{r}\sqrt{r^{2}-y^{2}}dy$$ (21)

The friction *T* of the bottom surface of the sliding body is

$$T=\left(S_{2}+S_{3}\right)c+N\;\text{tan}\;\varphi$$ (22)

(3) The friction *T'* can be calculated using the following formula:

$$T^{\prime }=\frac{r^{2}c}{2\;\text{tan}\;\alpha }+\frac{r^{2}K{\sigma \prime }_{z}\;\text{tan}\;\varphi }{2\;\text{tan}\;\alpha }$$ (23)

where ${\sigma^{\prime }}_{z}=\frac{2\sigma_{v}+r\gamma^{\prime }}{3}$ is the average vertical stress of the cone-like sliding body[36].

A force balance analysis for the sliding body is carried out. The horizontal force balance of the sliding body is

$$T\;\text{cos}\;\alpha +2T^{\prime }\;\text{cos}\;\alpha =N\;\text{sin}\;\alpha +F_{x}$$ (24)

The vertical force balance of the sliding body is

$$P_{v}+G=T\;\text{sin}\;\alpha +2T^{\prime }\;\text{sin}\;\alpha +N\cos\alpha$$ (25)

The required minimum horizontal seepage force *F*_*x*_ can be obtained by combining Eqs (24) and (25).

$$F_{x}=\frac{3\pi r^{2}c+4T^{\prime }\;\text{sin}\;\alpha }{2\;\text{sin}\;\alpha \left(\;\text{cos}\;\alpha +\;\text{sin}\;\alpha \;\text{tan}\;\varphi \right)}-\frac{\;\text{sin}\;\alpha -\;\text{cos}\;\alpha \;\text{tan}\;\varphi }{\;\text{cos}\;\alpha +\;\text{sin}\;\alpha \;\text{tan}\;\varphi }\left(P_{v}+G\right)$$ (26)

The seepage force is converted to the critical hydraulic gradient by combining the seepage force from Eq (15) and the seepage flow path *r*/tan *α* of the modelas follows:

$$J=\frac{3\pi r^{2}c+4T^{\prime }\;\text{sin}\;\alpha }{\left(\pi +4\right)r^{3}\gamma_{w}\;\text{cos}\;\alpha \left(\;\text{sin}\;\alpha \;\text{tan}\;\varphi +\;\text{cos}\;\alpha \right)}-\frac{2\;\text{tan}\;\alpha \left(P_{v}+G\right)(\;\text{sin}\;\alpha -\;\text{cos}\;\alpha \;\text{tan}\;\varphi )}{\left(\pi +4\right)r^{3}\gamma_{w}(\;\text{sin}\;\alpha \;\text{tan}\;\varphi +\;\text{cos}\;\alpha )}$$ (27)

The cohesion and internal friction angle are both constant, for a specified soil type, which simplifies the formulaabove to

$$J=\frac{A}{r}-B$$ (28)

where A and B are parameters related to the cohesion and internal friction angle of the soil.

## 4. Analysis and discussion

### 4.1 Effects of overburden pressure

**Fig 15:**
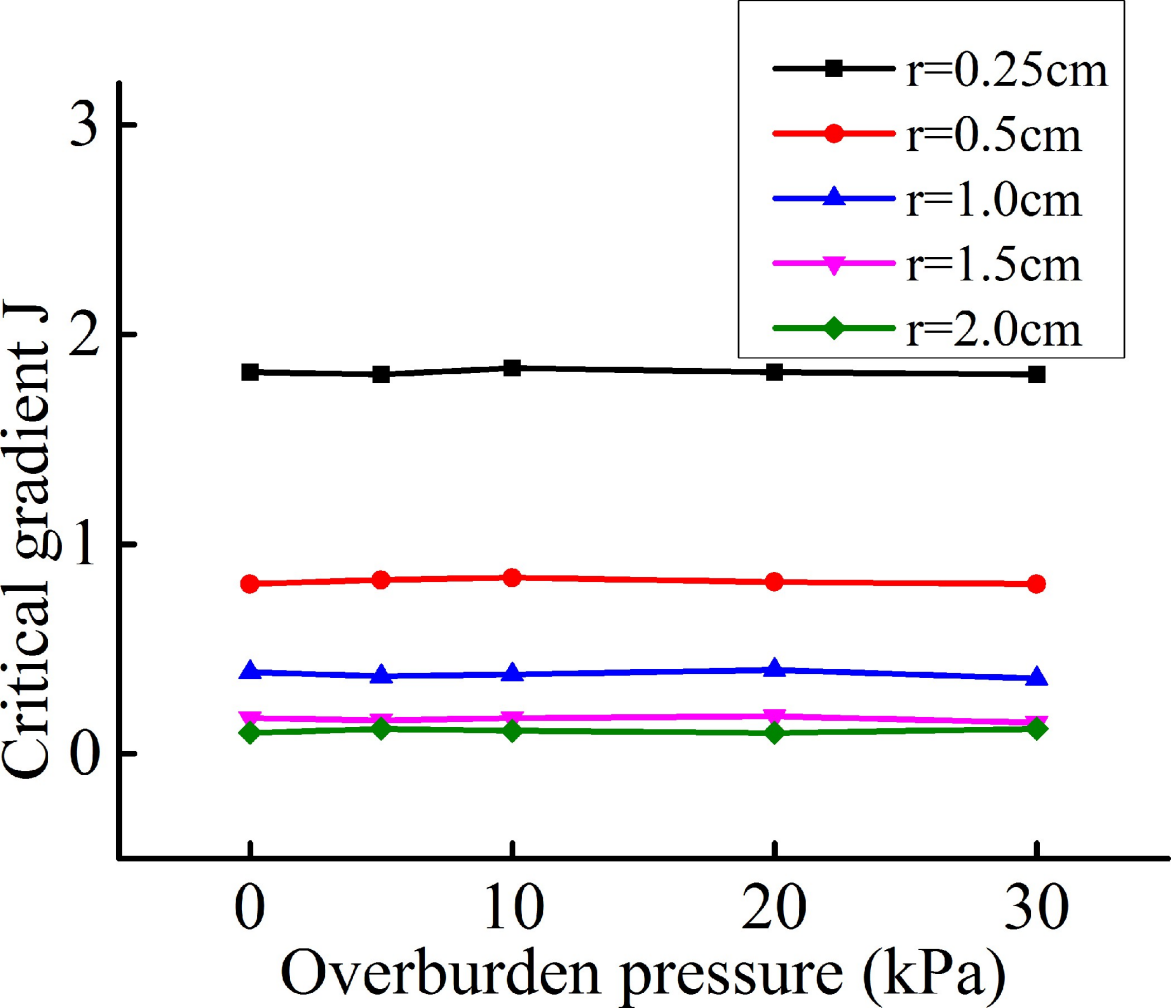
Variation trends of the critical gradient with the overburden pressure.

Fig 15 shows the variation trends of the critical gradient with the overburden pressure for Soil A and different hole radii. The data distribution shows that for a fixed hole radius, the critical gradient does not change as the overburden pressure increases. That is, the critical gradient is independent of the overburden pressure. Combining this result with Eq (27) shows that the effect of the overburden pressure on the stability of the structure mainly depends on the magnitude of *P*_*v*_. The calculation of the vertical force in Section 2.1 shows that when the soil thickness is much larger than the hole radius, a complete soil arch can form under the action of cohesion and the internal friction angle. The overburden pressure can then be neglected. Therefore, the overburden pressure has no effect on the critical gradient. The experimental results and theoretical results are in excellent agreement.

### 4.2 Effects of damage hole size

**Fig 16:**
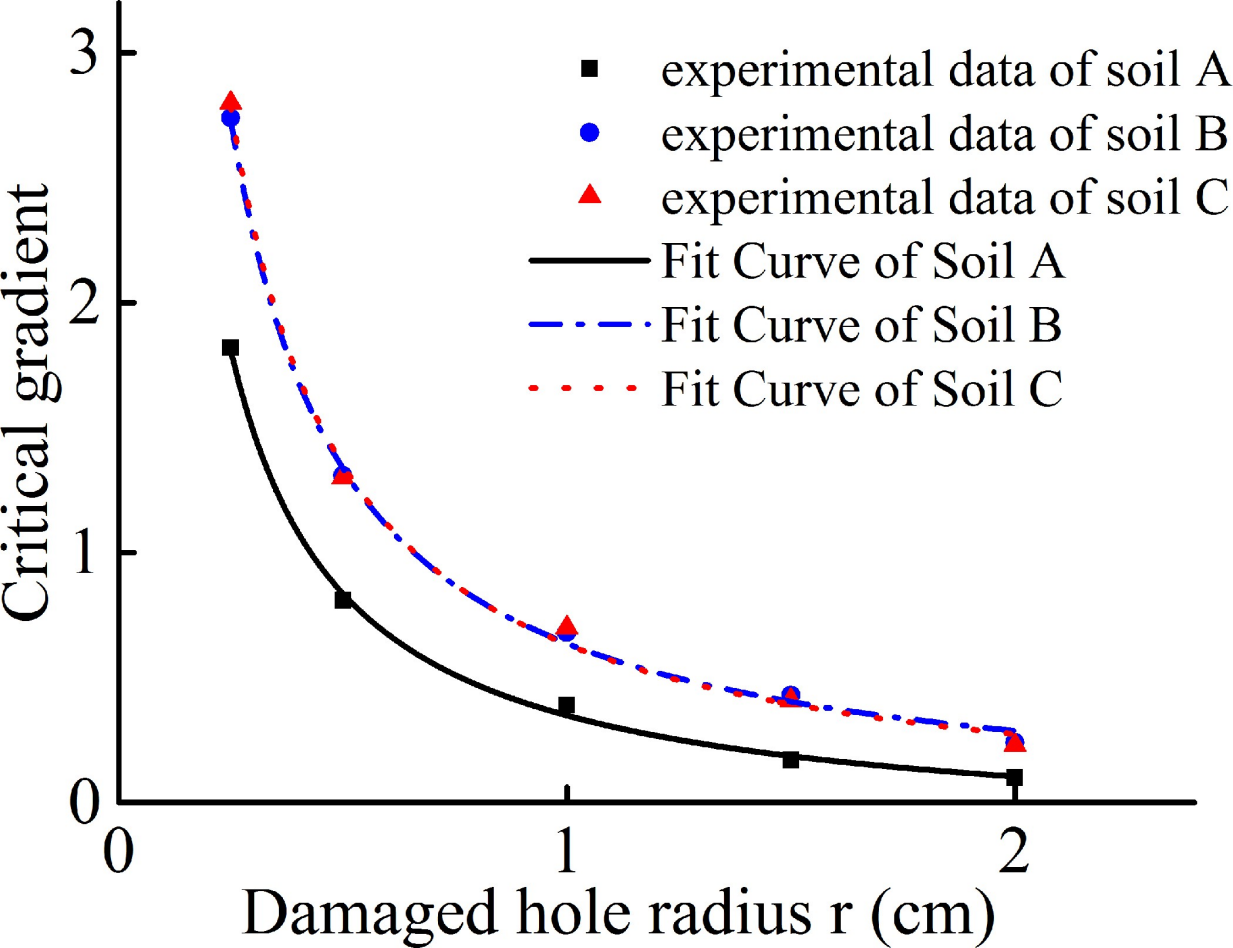
Fitting curves for variation in critical gradient with hole radius.

As shown in Fig 16, the experimentally obtained critical gradients under different working conditions are fitted using the model expression. The theoretical predictions are in excellent agreement with the experimental results.

As the hole radius increases, the critical gradient decreases at a nonlinear rate. When the hole radius is less than 1.0 cm, the critical gradient decreases sharply with the increasing hole radius. Upon further increase in the hole radius, the critical gradient continues to decrease, but the rate of decrease is significantly reduced.

### 4.3 Effects of internal friction angle and cohesion

Soils B and C have the same cohesion but different internal friction angles. Fig 16 shows only a small difference between the experimentally obtained critical gradients for the two soils, and coincidence of the corresponding theoretical fitting curves. Therefore, for a soil with a certain cohesion, the internal friction angle has a relatively small effect on the critical gradient. This result is obtained because increasing the internal friction angle increases the soil shear strength while reducing the lateral pressure coefficient, thereby weakening the soil arch effect.

Soil A has a much lower cohesion and corresponding critical gradient than Soils B and C. Therefore, soil cohesionhas a more significant effect on the critical gradient than the internal friction angle.

## 5. Conclusion

The mechanisms for horizontal seepage failure from damage to a geotube sidewall were studied by performing laboratory tests and developing a mathematical model. The effects of the overburden pressure, the hole radius and the soil properties were considered. The study conclusions are given below.

Soil A has a high coarse particle content and low cohesion. The erosion process for this soil inside the damaged tube is classified into three stages: stable seepage, initial soil production, and cyclic soil production. (1) In the stable stage, seepage erosion is negligible, and there is no visible specimen deformation. (2) During initial soil production, soil particles start to wash out through the damaged hole. Most of the soil washed out accumulates at the hole and prevents further soil production, thereby inhibiting the development of seepage failure. (3) During cyclic soil production, an incremental increase in the upstream hydraulic head results in a characteristic soil structural cycle of production, stability and re-production until a semicircular collapse pit forms at the top of the soil. Soils B and C have a higher silt content and cohesion than Soil A. Thus, for these two soils, most of the soil washed out is suspended in the water-outlet chamber before flowing out. The soil washed out is insufficient to submerge the hole and thereby hinders the soil washing out. After initial soil production begins, seepage erosion does not terminate. Due to complete soil arching effect, a horizontal soil flow channel forms finally in the soil.

The critical gradient is defined as the hydraulic gradient between the downstream section of the seepage field and the water-outlet chamber when soil starts to wash out. The experimentally obtained critical gradients were fitted with the developed mathematical model. The excellent agreement between the predicted and experimental results indicates the high reliability and practical value of the model.

The experimental and theoretical results were used to deduce the influence factors for the critical gradient. The critical gradient is independent of the overburden pressure and is weakly affected by the internal friction angle. The critical gradient increases with the cohesion. For fixed cohesion, small variations in the soil internal friction angle have a negligible influence on the critical gradient. Holding the other characteristic parameters fixed, the critical gradient decreases as the radius of the damaged hole increases. Moreover, as the hole radius increases, the rate of decrease of the critical gradient is gradually reduced.

## 6. Scope and limitations

The results of this study are applicable to horizontal seepage failure of soil in a damaged sidewall of the seam between geotube dam tubes. The model can also describe seepage failure when damaged geotextiles are used as a filter for vertical slopes. Contact erosion and scour of soil in joints channel subjected to seepage flow were not considered here and will be investigated in future research.
